# Predicting 30-days mortality for MIMIC-III patients with sepsis-3: a machine learning approach using XGboost

**DOI:** 10.1186/s12967-020-02620-5

**Published:** 2020-12-07

**Authors:** Nianzong Hou, Mingzhe Li, Lu He, Bing Xie, Lin Wang, Rumin Zhang, Yong Yu, Xiaodong Sun, Zhengsheng Pan, Kai Wang

**Affiliations:** 1 Department of Hand and Foot Surgery, Zibo Central Hospital, Shandong First Medical University, Zibo, 255036 Shandong China; 2 Independent researcher, bs20m2l@leeds.ac.uk, Leeds, LS29JT UK; 3 Institute of Medicine and Nursing, Hubei University of Medicine, Shiyan, 442000 Hubei China; 4 Department of Critical Care Medicine, Zibo Central Hospital, Shandong First Medical University , Zibo, 255036 Shandong China; 5 Fengnan District Maternal and Child Health Care Hospital of Tangshan City, Tangshan, 063300 Hebei China; 6 Department of Urology Surgery, Zibo Central Hospital, Shandong First Medical University , Zibo, 255036 China

**Keywords:** MIMIC-III, Sepsis-3, Machine learning, Xgboost, Logistic regression, SAPS-II score

## Abstract

**Background:**

Sepsis is a significant cause of mortality in-hospital, especially in ICU patients. Early prediction of sepsis is essential, as prompt and appropriate treatment can improve survival outcomes. Machine learning methods are flexible prediction algorithms with potential advantages over conventional regression and scoring system. The aims of this study were to develop a machine learning approach using XGboost to predict the 30-days mortality for MIMIC-III Patients with sepsis-3 and to determine whether such model performs better than traditional prediction models.

**Methods:**

Using the MIMIC-III v1.4, we identified patients with sepsis-3. The data was split into two groups based on death or survival within 30 days and variables, selected based on clinical significance and availability by stepwise analysis, were displayed and compared between groups. Three predictive models including conventional logistic regression model, SAPS-II score prediction model and XGBoost algorithm model were constructed by R software. Then, the performances of the three models were tested and compared by AUCs of the receiver operating characteristic curves and decision curve analysis. At last, nomogram and clinical impact curve were used to validate the model.

**Results:**

A total of 4559 sepsis-3 patients are included in the study, in which, 889 patients were death and 3670 survival within 30 days, respectively. According to the results of AUCs (0.819 [95% CI 0.800–0.838], 0.797 [95% CI 0.781–0.813] and 0.857 [95% CI 0.839–0.876]) and decision curve analysis for the three models, the XGboost model performs best. The risk nomogram and clinical impact curve verify that the XGboost model possesses significant predictive value.

**Conclusions:**

Using machine learning technique by XGboost, more significant prediction model can be built. This XGboost model may prove clinically useful and assist clinicians in tailoring precise management and therapy for the patients with sepsis-3.

## Background

Sepsis is a common and economically significant disease which has become an important public health issue globally and led to over 5.3 million people dies annually with an approximately overall mortality of 30%, particularly in the intensive care unit (ICU) [1–3]. Sepsis is defined as a syndrome of physiologic, pathologic, and biochemical abnormalities induced by infection which results in life-threatening organ dysfunction caused by dysregulated host response [3]. Different from those previous diagnostic criteria for sepsis, sepsis-3 highlighted the strong association between infection and organ failure according to the Third International Consensus Definitions for Sepsis and Septic Shock in February 2016 [2], hence, the early identification and diagnosis for sepsis are essential, which could provide meaningful information for clinicians to assess patients' condition and improve survival outcomes through prompt and appropriate treatment. Due to the complex of vague sepsis syndrome definitions, unknown sources of infection and higher mortality, it is necessary to establish a reliable and effective prognostic model for sepsis. With the help of these prognostic models, strong evidences for clinical decision-making and rational allocation of public health care resources can be provided.

The establishment of prognosis model for sepsis patients has always been a hot topic in critical care medicine. Some sensitive serum markers, such as Ang-2, PCT, interleukin-6, pentraxin 3, etc. [1, 4, 5], have been widely used to facilitate sepsis prognosis, however, their prognostic values are limited, not only rarely available but often lack of sensitivity or specificity. On the other hand, traditional prediction models based on small sample data such as logistic regression analysis and scoring systems including acute physiology and chronic health evaluation-II (APHACHE-II), Simplified acute physiology score-II (SAPS-II) and etc. [6–8], are still providing comprehensively clinical importance of identifying patients who are at risk of unfavourable prognostic outcomes, but these methods and scores require the statistical assumption of the independent and linear relationship between explanatory and outcome variables or preclude the analysis of a large number of valuable variables. In addition, insufficient prognostic strength, large fluctuation range, poor stability and operability, tedious process, and other shortcomings exist in these predictive serum markers, models and scores to a certain extent.

Recently, novel machine learning techniques have demonstrated improved predictive performance compared to traditional prediction methods. Moreover, the evolution of statistical theory, computer technology and the establishment of specialized database for critical care medical such as MIMIC-III could help machine learning get more attention and recognition by clinicians. eXtreme Gradient Boosting (XGBoost) is a machine learning technique with the remarkable features of processing the missing data efficiently and flexibly and assembling weak prediction models to build a accurate one [9]. As an open source package, XGBoost has been widely recognized in a number of machine learning and data mining challenges, for example, 17 solutions used XGBoost among the 29 challenge winning solutions published at Kaggle’s blog in 2015 and the top-10 winning teams used XGBoost in KDD Cup 2015 [10].

Therefore, the goal of the study was twofold: firstly, we attempted to compare the performance of machine learning (XGboost) model with traditional prediction models (conventional logistic regression model and SAPS-II score model) in the prediction of the 30-days mortality in MIMIC-IIIpatients with sepsis-3. Secondly, we planned to plot nomogram and clinical impact curve (CIC) to validate the XGboost model.

## Methods

### Database

We used the Medical Information Mart for Intensive Care III database version 1.4 (MIMIC III v1.4) for the study. MIMIC-III, a publicly available single-center critical care database which was approved by the Institutional Review Boards of Beth Israel Deaconess Medical Center (BIDMC, Boston, MA, USA) and the Massachusetts Institute of Technology (MIT, Cambridge, MA, USA), includes information on 46,520 patients who were admitted to various ICUs of BIDMC in Boston, Massachusetts from 2001 to 2012 [11–13]. The database contains charted events such as demographics, vital signs, laboratory tests, fluid balance and vital status; documents International Classification of Diseases and Ninth Revision (ICD-9) codes; records hourly physiologic data from bedside monitors validated by ICU nurses; and stores written evaluations of radiologic films by specialists covering in the corresponding time period. The use of the data in the database, provided by clinicians, data scientists, and information technology personnel and unidentified health information of patients, has been deemed not human subjects research and there was no requirement for individual patient consent because of the unidentified health information [12, 13]. The users, whereas, must pass a test to qualify to register for the database and be approved by MIMIC-III database administration staff. After passing a training course “Protecting Human Research Participants” on the website of National Institutes of Health (NIH), an author (NZ Hou) was approved to extract data from this database for research purposes (certification number: 37258322).

### Study population

Adult patients who were diagnosed with sepsis-3 were included in our study. The inclusion criteria were: (I) patients who were older than 18 years old; (II) length of stay in the ICU was over 24 h to ensure sufficient data for analysis; (III) patients with the diagnosed of sepsis according to The Third International Consensus Definitions for Sepsis and Septic Shock (sepsis-3) [2]. Because MIMIC-III database has shifted their date of birth to obscure their age, we excluded patients who were over 89 years, and if a patient had multiple admissions with sepsis, only the first admission was analyzed. As it is common with missing data in the MIMIC-III database, we also removed the variables with more than 20% observations missing to facilitate and ensure the accuracy of the review. However, for those with less than 20% missing data or randomly missing data, we explored and visualized them with Templ’s method (R Package “VIM”) [14] and multiple imputation method (R Package “mice”) [15] for further analysis respectively.

### Data extraction

**Fig. 1:**
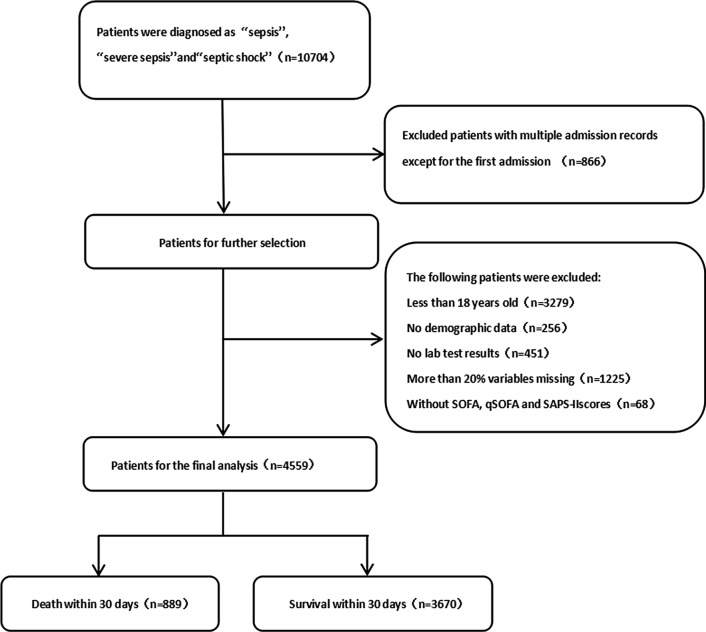
The detailed process of data extraction

We obtained the raw data about patients who were diagnosed with “sepsis”, “severe sepsis” and “septic shock” on discharge using pgAdmin PostgreSQL tools (version 1.22.1) and Navicat Premium (version 12.0.28). After that, R software (version 3.4.3, CRAN) was used for further process. The code, supporting the MIMIC-III documentation and generating the descriptive statistic, is publicly available and contributions from the community of users are encouraged (https://github.com/MIT-LCP). The detailed process of data extraction is shown in Fig. 1. Following demographic data were extracted: age, gender, ethnicity, weight, height and body mass index (BMI), length of stay in hospital, length of stay in the ICU, hospital expire flag (in-hospital death recorded in the hospital database) at the first ICU admission. Then, we collected vital signs of the patients from the first 24 h of ICU stay, including heart rate (HR), systolic blood pressure (SBP), diastolic blood pressure (DBP), mean arterial pressure (MAP), temperature (TEMP), respiratory rate (RR) and oxyhemoglobin saturation (SpO2). Afterwards, laboratory values, such as blood routine examination, liver and kidney function, blood glucose, and arterial blood gas (ABG) were abstracted. Furthermore, advanced cardiac life support (mechanical ventilation, renal replacement therapy, etc.) and accompanied diseases (diabetes, malignant tumour, etc.) were accessed. Because of the high sampling frequency, we use the maximum, minimum and the mean value when incorporating the characteristics of vital signs and related laboratory indicators. Ultimately, we obtained the list of anonymized patients with sepsis from the Table 1 (Additional file 1).

**Table 1:** Baseline characteristics, vital signs, laboratory parameters and statistic results of mimic-III patients with sepsis

	Death within 30 days	Survival within 30 days	*p*
Number (sample size)	889	3670	
Baseline variables and in-hospital factors			
Age (year, mean SD)	71.42 ± 15.93	63.61 ± 17.73	8.95E−36
Sex (%)			
Female	413	1609	
Male	476	2061	0.1706
Ethnicity (%)			
White	617 (69.4%)	2642 (72.1%)	
Black	66 (7.4%)	338 (9.2%)	
Yellow	34 (3.8%)	145 (4%)	
Others	172 (19.3%)	545 (14.9%)	0.005914
Weight (kg), mean (SD)	76.91 ± 21.31	82.69 ± 28.61	
Height (cm), mean (SD)	168.27 ± 11.66	169.51 ± 10.73	
BMI (kg/m^2^), mean (SD)	27.82 ± 7.65	29.25 ± 8.91	
Length of stay in hospital, days, mean (SD)	7.04 ± 6.3	10.99 ± 10.4	
Length of stay in the ICU, days, mean (SD)	4.87 ± 4.99	4.66 ± 6.31	
Admission type			
MED	580 (65.2%)	1912 (52.1%)	
CMED	86 (9.7%)	477 (13.0%)	
Others	223 (25.1%)	1282 (34.9%)	1.36E−11
Vital signs			
Heartrate_min (times/min), mean (SD)	72.92 ± 19.16	73.08 ± 15.72	0.826205355
Heartrate_mean (times/min), mean (SD)	91.12 ± 18.27	87.81 ± 16.15	0.00000579
Sysbp_min (mmhg), mean (SD)	80.46 ± 20.01	90.69 ± 16.18	6.49E−36
Diasbp_mean (mmhg), mean (SD)	58.65 ± 10.42	61.7 ± 10.08	6.62E−13
Meanbp_min (mmhg), mean (SD)	48.41 ± 15.65	56.24 ± 13.89	6.74E−34
Resprate_mean (times/min),mean (SD)	21.73 ± 4.68	19.54 ± 4.06	3.66E−30
Tempc_min (℃), mean (SD)	35.77 ± 1.17	36.15 ± 0.86	1.78E−16
Tempc_max (℃), mean (SD)	37.34 ± 1.17	37.65 ± 0.85	3.37E−11
Spo2_mean (%), mean (SD)	96.03 ± 4.02	97.1 ± 1.96	3.06E−12
Laboratory parameters			
Aniongap_max (mmhg), mean (SD)	19.12 ± 6.26	16.17 ± 4.65	1.45E−31
Aniongap_min (mmhg), mean (SD)	14.58 ± 4.66	12.45 ± 3.08	1.48E−30
Creatinine_min (ng/dL), mean (SD)	1.65 ± 1.23	1.35 ± 1.39	3.89E−09
Chloride_min (mmol/L), mean (SD)	101.67 ± 7.65	101.93 ± 6.69	0.39868745
Hemoglobin_min (g/dL), mean (SD)	9.84 ± 2.21	10.08 ± 2.08	0.009534544
Hemoglobin_max, (g/dL), mean (SD)	11.77 ± 2.26	12 ± 2.09	0.012634047
Lactate_min (mmol/L), mean (SD)	2.36 ± 2.07	1.55 ± 0.81	4.26E−24
Platelet_min (10^9^/L), mean (SD)	189.73 ± 125.29	195.98 ± 108.29	0.207608419
Potassium_min (mmol/L), mean (SD)	3.84 ± 0.7	3.71 ± 0.54	0.00000328
Sodium_min (mmol/L), mean (SD)	136.27 ± 6.62	136.08 ± 5.35	0.454879474
Sodium_max (mmol/L), mean (SD)	141.28 ± 6.8	140.51 ± 5.03	0.003570629
Bun_min (mmol/L), mean (SD)	36.09 ± 25.43	24.22 ± 19.69	8.45E−31
Bun_max (mmol/L), mean (SD)	42.88 ± 28.49	30.23 ± 24.39	1.12E−27
Wbc_min (10^9^/L), mean (SD)	12.54 ± 12.22	10.41 ± 6.55	4.81E−06
Wbc_max (10^9^/L), mean (SD)	17.54 ± 19.99	14.8 ± 9.87	0.000293182
Inr_max, mean (SD)	2.12 ± 1.79	1.61 ± 1.34	2.89E−14
Urine output	1225.29 ± 1307.53	1993.04 ± 1551.57	2.82E−48
Score system			
SOFA	8.02 ± 4.33	5.22 ± 2.85	2.74E−55
qSOFA	2.15 ± 0.64	1.9 ± 0.69	4.60E−21
SAPS II	54.67 ± 16.37	37.51 ± 13.22	2.2E−16
Advanced life support			
Mechanical ventilation	531 (59.69%)	1668 (45.55%)	
Renal replacement therapy	53 (5.95%)	135 (3.61%)	0.2503
Accompanied diseases (comorbidity)			
Diabetes	246 (27.64%)	2631 (71.70%)	
Malignant tumour	116 (13.05%)	160 (4.37%)	
Others	527 (59.31%)	879 (23.93%)	2.20E−16
Common sources of infection			
Blood culture	418 (47%)	1343 (36.6%)	
MRSA screen	267 (30%)	1384 (37.72%)	
Urine	151 (17%)	642 (17.5%)	
Swab	18 (2%)	70 (1.9%)	
Others	35 (4%)	231 (6.3%)	7.82E−08
Outcome			
Within 30-days mortality	19.50%	80.50%	

*MED* medical-general service for internal medicine, *CMED* cardiac medical-for non-surgical cardiac related admissions, *sysbp* systolic blood pressure, *diasbp* diastolic blood pressure, *meanbp* mean blood pressure, *resprate* respiratary rate, *tempc* temperature, *bun* blood urea nitrogen, *wbc* white blood cell, *INR* international normalized ratio, *sofa* sequential organ failure assessment, *qSOFA* quick SOFA, *SAPS II* simplified acute physiology score II, *Spo2* oxyhemoglobin saturation, *Max* maximum, *Min* minimum

### Statistical analysis

Patients were divided into two groups based on whether death or alive within 30 days and variables were displayed and compared between groups. We revealed and excluded these confounders of the independent risk factors, then, performed correlation analysis to determine the impact of them on 30-days mortality. Normally and non-normally distributed continuous variables were summarized as the mean ± SD and the median respectively. Continuous variables of normal distribution were tested by Kolmogorov–Smirnov test. Student’s t test, One-way ANOVA, Mann–Whitney U or Kruskal–Wallis H test were used to compare continuous data of non-normally distribution, if appropriate. Categorical variables were expressed as numbers or percentage and assessed using Chi-square test or Fisher’s exact test according to different sample sizes as proper.

In the model-development phase, we constructed three predictive models: conventional logistic regression model, SAPS-II score model and XGBoost algorithm model. Firstly, the conventional logistic regression model was conducted using these significant variables identified by backward stepwise analysis with Chi-square test. Then we chose an entry probability of < 0.05 by the stepwise selection method. Secondly, in the construction of SAPS II model, we used these time-stamp variables to do prediction based on the methods provided by the original literature of SAPS II [16]. Thirdly, we performed XGBoost model [17, 18] to analysis the contribution (gain) of each variable to 30-days mortality, at the same time, backward stepwise analysis was processed to select the variable with a threshold of p < 0.05 according to the Akaike information criterion (AIC) [19]. After identifying the variables through XGBoost, we used these clinical and laboratory variables included to construct the XGBoost algorithm model. In the model-comparison phase, we tested and compared the performances of the three predictive models by area under curves (AUCs) of the receiver operating characteristic curves (ROC) and decision curve analysis (DCA), then, selected the model that achieved the highest overall diagnostic value for further verification. At last, nomogram and clinical impact curve (CIC) were plotted to evaluate the clinical usefulness and applicability net benefits of the model with the best diagnostic value. All the analyses above were conducted using R software, and *p* value < 0.05 was defined as statistically significant.

## Results

### Baseline characteristics

**Fig. 2:**
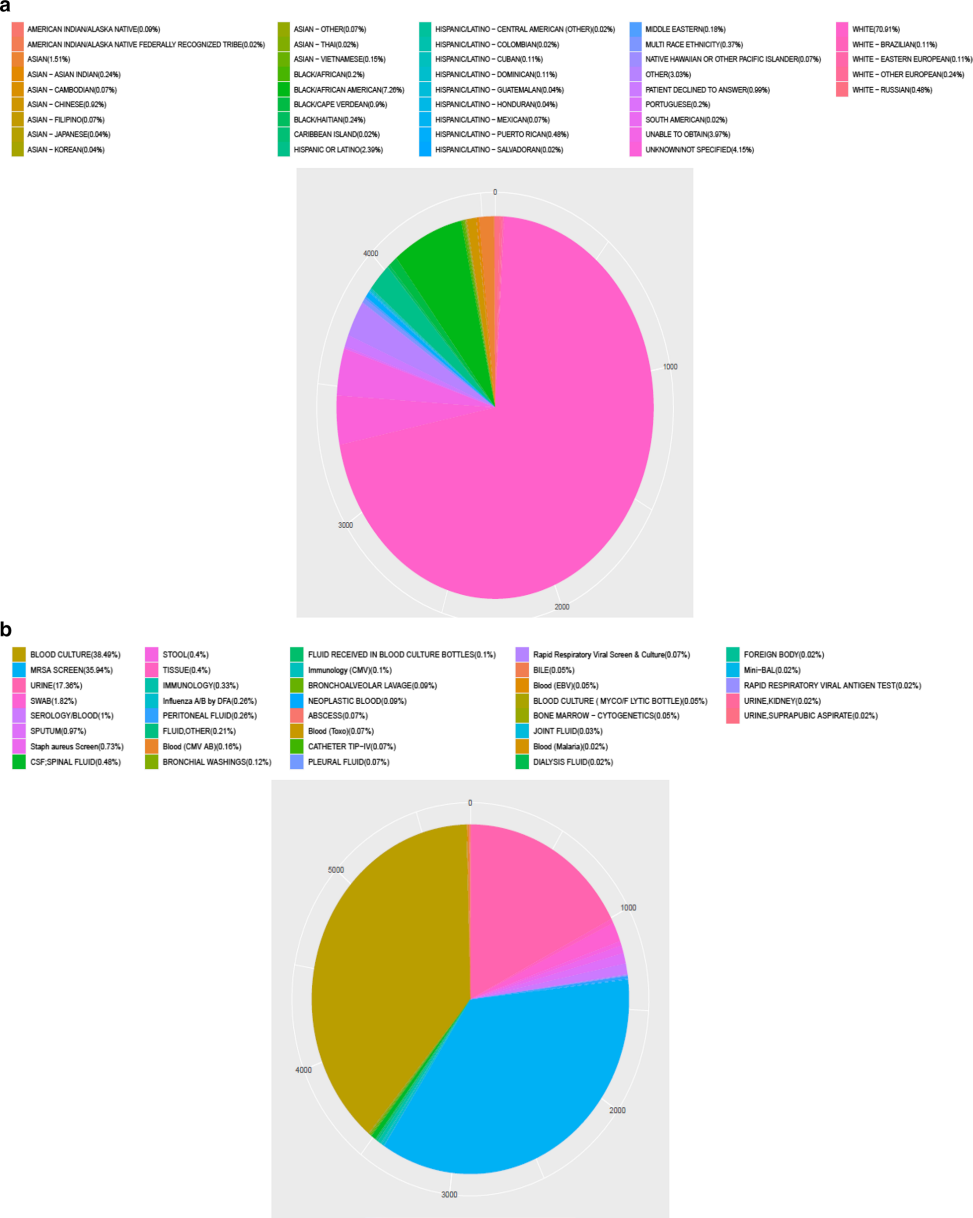
Characteristics of MIMIC-III patients with sepsis by ethnicities (**a**) and characteristics of MIMIC-III patients with sepsis by common sources of infection (**b**)

A total of 4559 sepsis-3 patients are included in our study, in which, 889 patients were death and 3670 survival within 30 days, respectively. In these patients of death, the age, ethnicity, admission type, heartrate_mean, sysbp_min, diasbp_mean, meanbp, meanbp_min, resprate_mean, tempc_min/max, spo2_mean, aniongap (AG)_min/max, creatinine_min, hemoglobin_min/max, lactate_min, potassium_min, sodium_max, bun (blood urea nitrogen)_min/max, wbc (white blood cell)_min/max/mean, INR (international normalized ratio)_max/mean, urine output, score system, comorbidity and common sources of infection differ significantly compared these of survived, however, the sex, heart rate_min, chloride_min, platelet_min, sodium_min and advanced life support show no significant difference between the two groups. Figure 1 is a flow chart describing the procedure for subjects selection; Table 1 is a summary concluding the comparisons of the baseline characteristics, vital signs, laboratory parameters between the non-survivors and the survivors within 30 days, and the overall ethnicity characteristics/the common sources of infection are listed in Fig. 2.

### Features selected in models

**Fig. 3:**
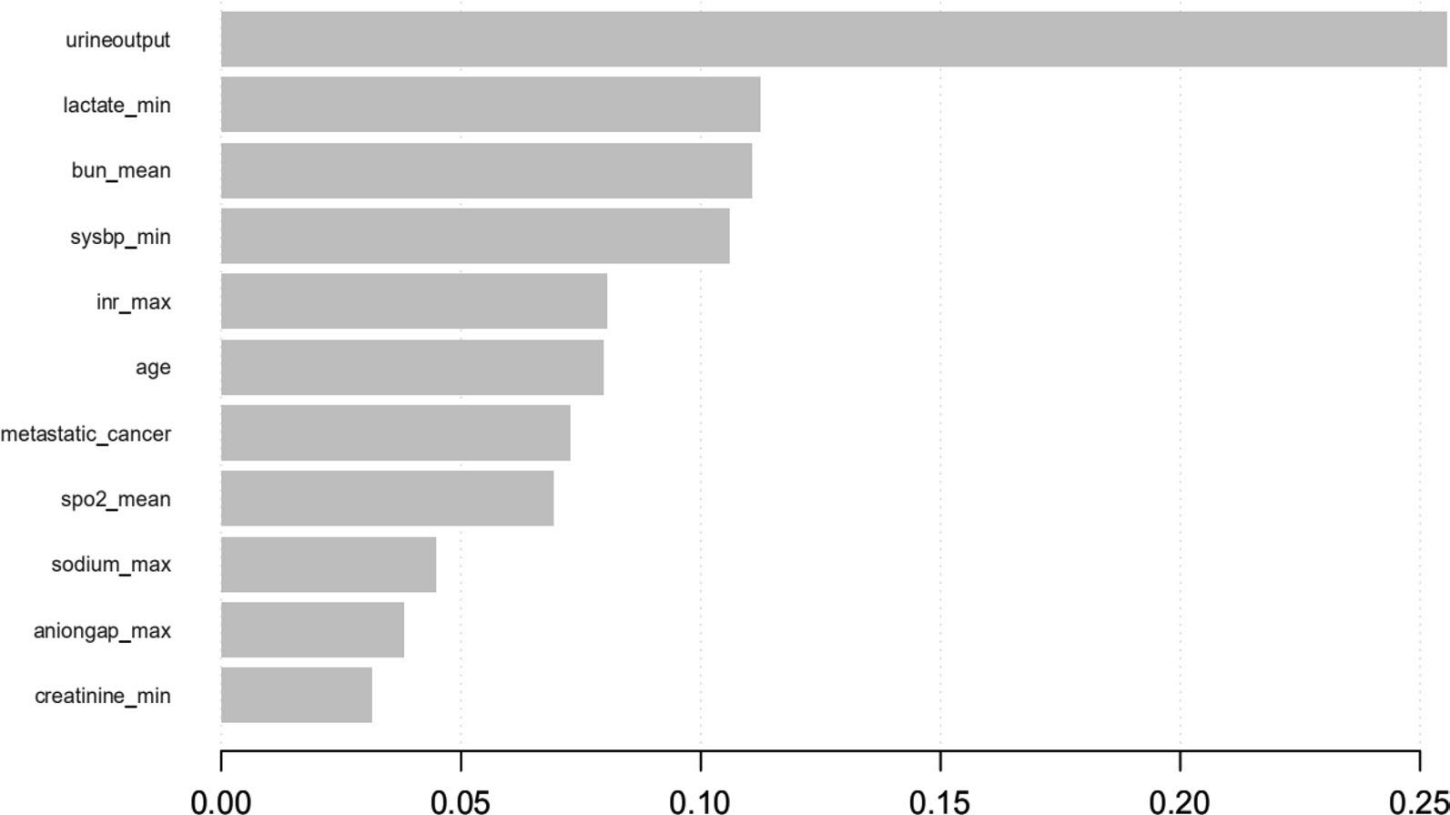
Top 11 features selected using XGBoost and the corresponding variable importance score. *X*-axis indicates the importance score which is the relative number of a variable that is used to distribute the data, *Y*-axis indicates the top 11 weighted variables

As shown in Table 2, the most important features, which were identified by the results of backward stepwise analysis and strongly associated with mortality in 30 days, were applied in conventional logistic regression model, all of which with *p* value < 0.05. Moreover, according to the analysis results of each features’ contribution by XGBoost model (Table 3 and Fig. 3), urine output, lactate, Bun, sysbp, INR, age, cancer, SpO2, sodium, AG, and creatinine were the top 11 most important features of the data set and these variables are also included to construct XGBoost predictive models in our study.

**Table 2:** Features selected in the conventional logistic regression

	OR_with_CI	p value
(Intercept)	52,913.003 (87.92–33,934,517.782)	< 0.001
Sofa	1.142 (1.106–1.179)	< 0.001
Aniongap_min	1.078 (1.043–1.115)	< 0.001
Creatinine_min	0.676 (0.592–0.767)	< 0.001
Chloride_min	0.98 (0.962–0.999)	0.03393
Hematocrit_min	1.113 (1.053–1.178)	< 0.001
Hemoglobin_min	0.748 (0.623–0.895)	0.00169
Hemoglobin_max	0.926 (0.863–0.992)	0.02993
Lactate_min	1.308 (1.194–1.435)	< 0.001
Potassium_min	1.179 (1.001–1.389)	0.04922
Sodium_max	1.046 (1.019–1.074)	< 0.001
Bun_min	1.033 (1.018–1.048)	< 0.001
Bun_max	0.986 (0.973–0.997)	0.01542
Wbc_min	1.062 (1.036–1.09)	< 0.001
Wbc_max	0.969 (0.952–0.987)	< 0.001
Heartrate_min	0.987 (0.977–0.997)	0.0111
Heartrate_mean	1.022 (1.011–1.033)	< 0.001
Sysbp_min	0.991 (0.984–0.998)	0.00839
Meanbp_min	0.992 (0.985–1)	0.0468
Resprate_mean	1.062 (1.038–1.086)	< 0.001
Tempc_min	0.897 (0.81–0.993)	0.03242
Tempc_max	0.781 (0.698–0.873)	< 0.001
Spo2_mean	0.947 (0.909–0.986)	0.00839
Age	1.029 (1.022–1.035)	< 0.001
Diabetes	0.779 (0.639–0.948)	0.01328
Vent	1.824 (1.48–2.251)	< 0.001

*OR* odds ratio, *CI* confidence interval, *SOFA* sequential organ failure assessment, *bun* blood urea nitrogen, *wbc* white blood cell, *sysbp* systolic blood pressure, *meanbp* mean blood pressure, *resprate* respiratary rate, *Spo2* oxyhemoglobin saturation, *vent* ventilation, *Max* maximum, *Min* minimum

**Table 3:** Features selected in the XGboost model

	OR_with_CI	*P*
(Intercept)	493.907 (9.063–27,931.087)	0.00247
Urineoutput	1 (1–1)	< 0.001
Lactate_min	1.401 (1.288–1.527)	< 0.001
Bun_mean	1.018 (1.013–1.023)	< 0.001
Sysbp_min	0.979 (0.974–0.984)	< 0.001
Metastatic_cancer	2.997 (2.217–4.038)	< 0.001
Inr_max	1.058 (1.002–1.115)	0.03709
Age	1.019 (1.013–1.025)	< 0.001
Sodium_max	1.016 (1.001–1.031)	0.03835
Aniongap_max	1.048 (1.026–1.069)	< 0.001
Creatinine_min	0.766 (0.686–0.852)	< 0.001
Spo2_mean	0.897 (0.865–0.93)	< 0.001

*OR* odds ratio, *CI* confidence interval, *bun* blood urea nitrogen, *sysbp* systolic blood pressure, *INR* international normalized ratio, *Spo2* oxyhemoglobin saturation, *Max* maximum, *Min* minimum

### Model comparisons

**Fig. 4:**
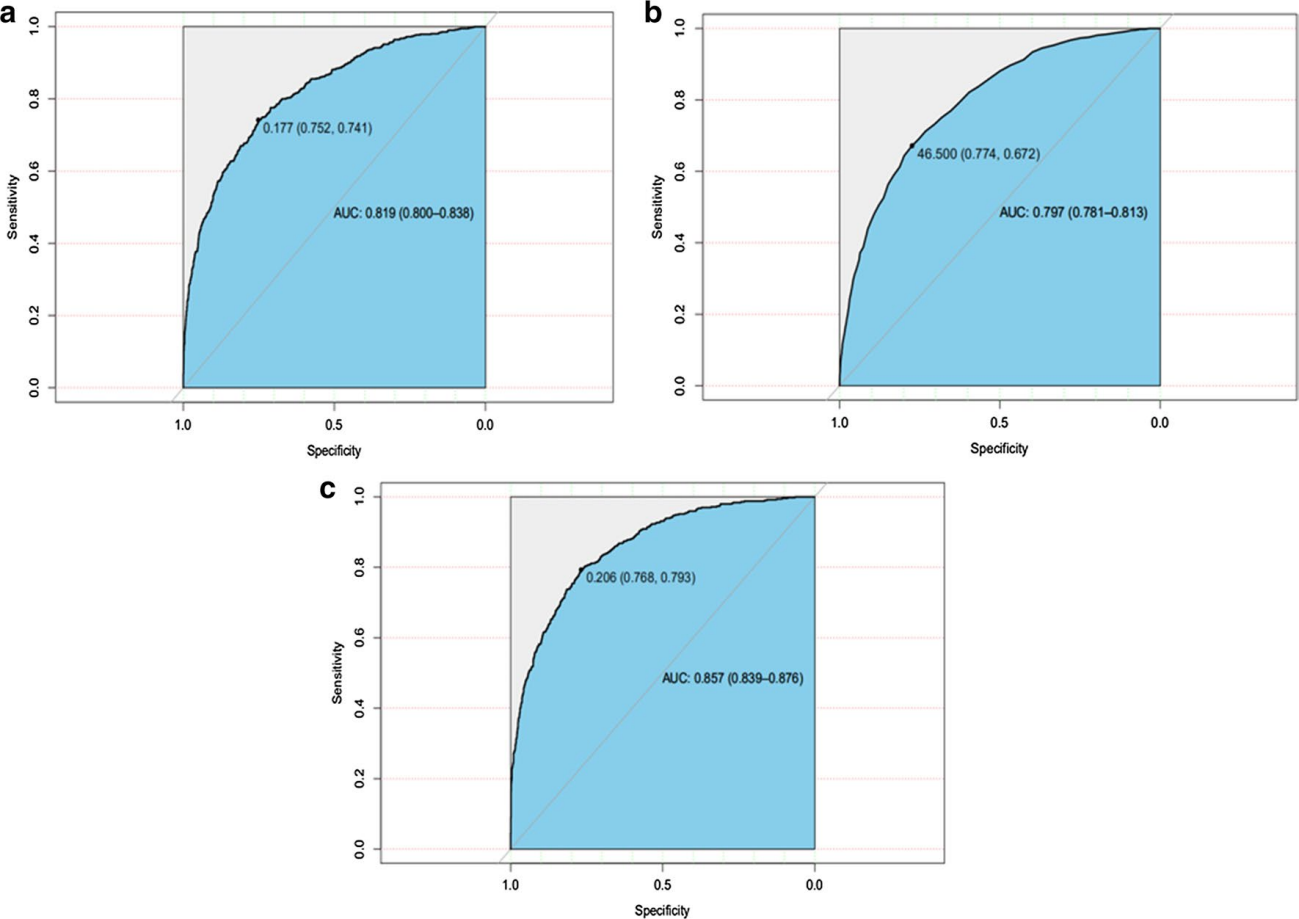
The receiver operating characteristic (ROC) curves. **a** traditional logistic regression model, area under curves (AUC) is 0.819 [95% confidence interval (CI); 0.800–0.838]; **b** SAPS-II score model, AUC is 0.797 [0.781–0.813]; **c** XGboost model, AUC is 0.857 [0.839–0.876], the best performance of the models was the XGboost model

**Fig. 5:**
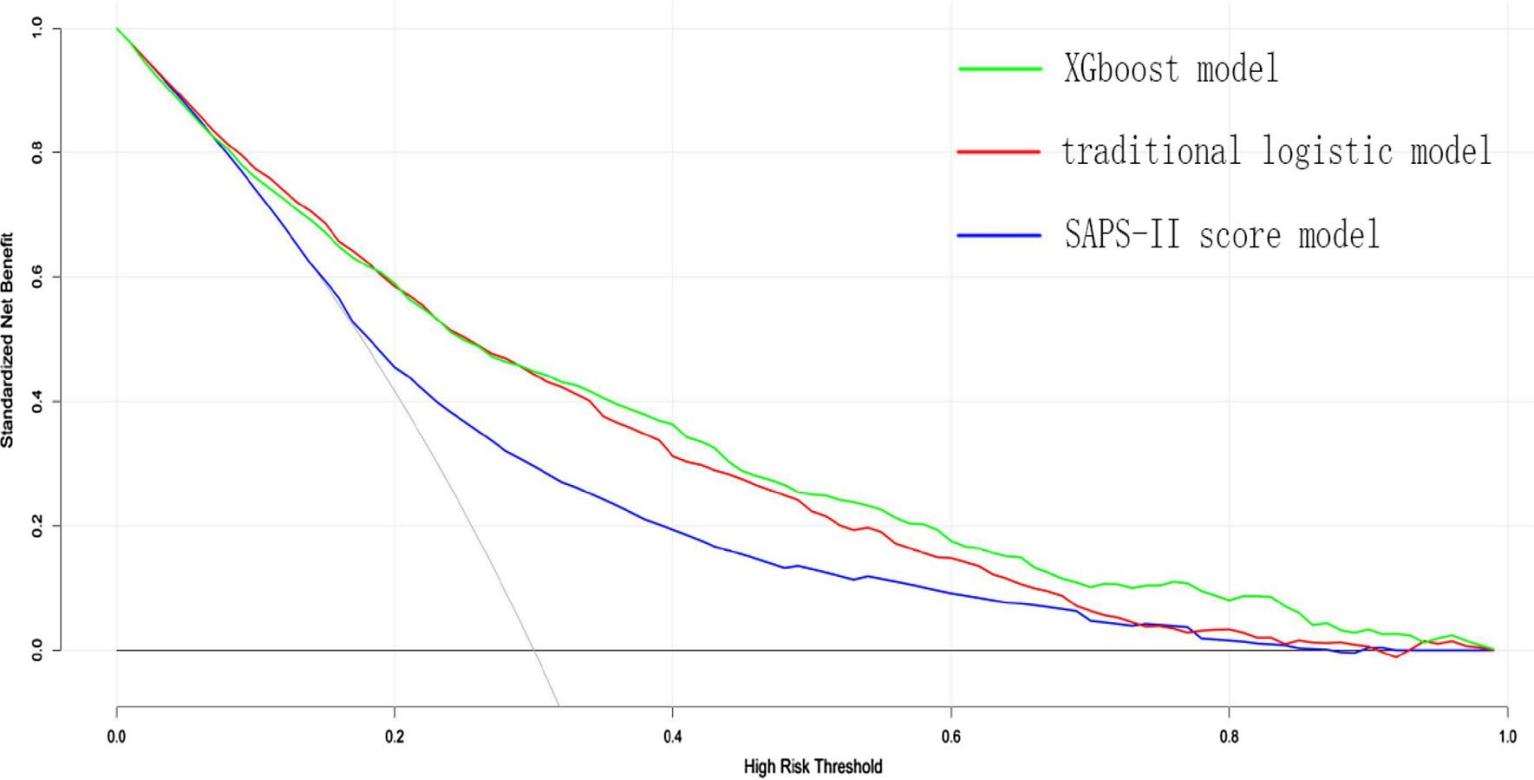
Decision curve analysis (DCA) of the three prediction models. The net benefit curves for the three prognostic models are shown. *X*-axis indicates the threshold probability for critical care outcome and *Y*-axis indicates the net benefit. Solid green line = XGboost model, solid red line = traditional logistic model, solid blue line = SAPS-II score mode. The preferred model is the XGboost model, the net benefit of which was larger over the range of traditional logistic model and SAPS-II score model

In the model-development and validation phase, the three models (traditional logistic regression model, SAPS-II score model and XGBoost algorithm model) showed good discriminatory power with AUCs of 0.819 (95% CI 0.800–0.838), 0.797 (95% CI 0.781–0.813), and 0.857 (95% CI 0.839–0.876), respectively (Fig. 4). The XGBoost algorithm model showed the largest test AUC but the traditional logistic regression model was the smallest. According to the DCA of the three prediction model, the net benefit for XGboost model was larger over the range of traditional logistic model and SAPS-II score model, which means XGboost model is the optimal and the SAPS-II score model inferior (Fig. 5).

### Optimal model analysis

**Fig. 6:**
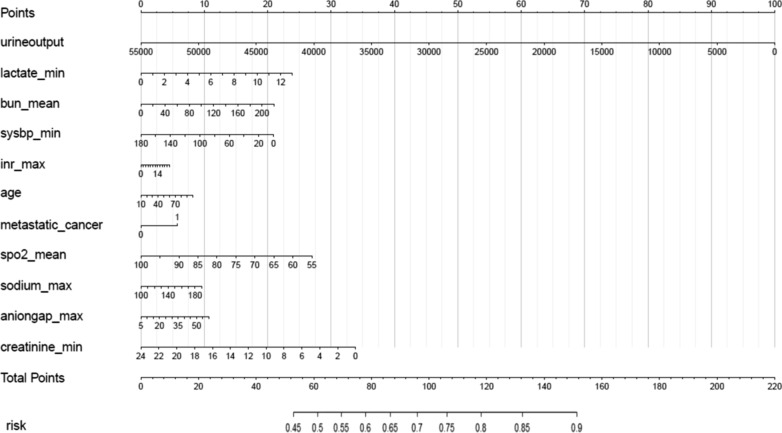
Nomogram to estimate the risk of mortality in sepsis patients. To use the nomogram, we first draw a line from each parameter value to the score axis for the score, the points for all the parameters are then added, finally, a line from the total score axis is drawn to determine the risk of mortality on the lower line of the nomogram

**Fig. 7:**
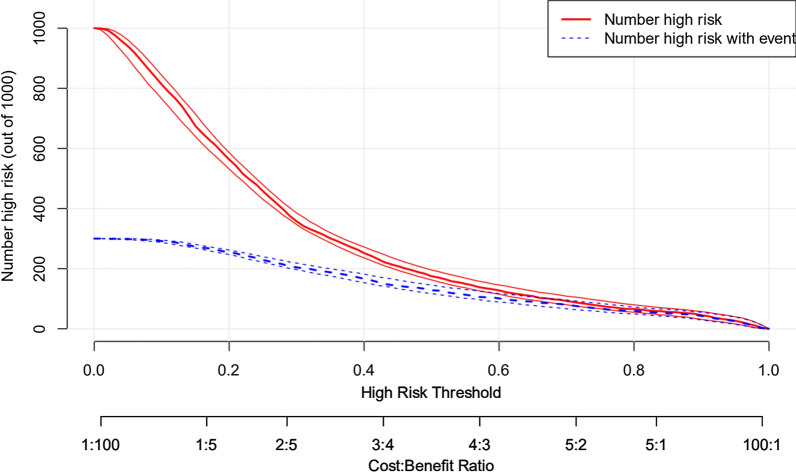
Clinical impact curve (CIC) of XGboost model. The red curve (number of high-risk individuals) indicates the number of people who are classified as positive (high risk) by the model at each threshold probability; the blue curve (number of high-risk individuals with outcome) is the number of true positives at each threshold probability. CIC visually indicated that nomogram conferred high clinical net benefit and confirmed the clinical value of the XGboost model

For visualization of the XGboost predictive model, the risk nomogram that integrated 11 selected variables for the incidence of mortality within 30 days is shown in Fig. 6. Clinical impact curve (CIC) analysis was performed in Fig. 7 to evaluate clinical applicability of risk prediction nomogram. CIC visually showed that the nomogram had a superior overall net benefit within the wide and practical ranges of threshold probabilities and impacted patient outcomes, which indicates that the XGboost model possesses significant predictive value.

## Discussion

Sepsis, which is associated with profound mortality and substantial economic burden, is no longer defined simply as serious infection. In a systematic review and meta-analysis, Reinhart et al. [20] concluded that the mortality rate estimate of ICU- and hospital-treated sepsis patients were 41.9% and 26.7% respectively, or one out of four sepsis patients did not survive their hospital stay. Torio et al. [21] estimated sepsis accounted for 6.2% of the aggregate costs for all hospitalizations, or 23.7 billion USD in 2011. Furthermore, Moss et al. [22] conducted a study spanning two decades (from 1979 to 2000), which reported the annual increase of sepsis cases was around 8.7%. The improvement of sepsis prevention, recognition, and treatment has been a global health priority since the declaration repeatedly by the World Health Organization (WHO) in 2017 [23]. Progressive exacerbation of sepsis can lead to organ failure and death, but early aggressive therapy also forestalls further progression and rescues a decompensating patient. Unfortunately, in ICU it is very difficult for clinicians to predict which patients will respond favorably and could be out of the crisis or will deteriorate despite all interventions and resuscitative efforts. At present, these findings indicate the urgent need to increase efforts to promote reliable prediction models to identify patients with sepsis who are at increased risk of developing organ dysfunction and to prognosticate their mortality.

In this present study, the AUCs and DCAs we developed have demonstrated the benefit of using a XGboost model- as opposed to the classic logistic regression analysis and traditional SAPS II scoring system for early prediction of probability of septic mortality. Moreover, CIC and nomogram were plotted to evaluate the clinical usefulness and applicability net benefits of the model with the best diagnostic value. Logistic regression analysis as one of the classic regression analyses is widely used to test the association between sepsis and mortality. For instance, through the logistic regression analysis, Vivien et al. [24] observed an association between mortality at day 28 and the tidal volume indexed on ideal body weight (VTIBW) in pre-hospital mechanically ventilated patients with septic shock; Wu et al. [25] revealed that dynamic changes of serum S100B levels from day 3 to 1 were more associated with mortality than those on day 1 in patients with sepsis; Oud et al. [26] indicated that sepsis was associated with most of the short-term deaths among ICU patients with SLE despite its relatively low mortality; Song et al. [5] revealed that combined biomarkers approach showed good performance in predicting 28-day all-cause mortality among patients diagnosed with either sepsis or septic shock according to the sepsis-3 definition, however, the differences might not be statistically proven. Furthemore, some studies [27, 28] found conventional logistic regression had a relatively low indicator of performance as measured by AUCs for ROC curves or showed higher prediction error and worsen performance compared to some novel techniques.

Several conventional prognostic scoring systems have been developed to provide relevant evaluation results considering the hospital mortality of ICU patients. The advantages of such scoring systems are easy to calculate and interpret. SAPS II, as one of the commonly used model, has better discrimination, calibration and power to predict deaths on ICU than the sequential organ failure assessment score (SOFA), which has been recommended for the identification and mortality prognostication of patients in ICU by sepsis-3 [7]. Moreover, the ability of SAPS II to discriminate between survivors and non-survivors is as excellent as APACHE II score and other scores and even to help to play in end-of-life decision-making in ICUs [8]. However, the specificity and sensitivity of scoring systems such as SAPS II are low, and the predictive performance is worse than that of multivariate predictive models. Last but not least, the evaluation systems and the accurate outcomes depended heavily on the practitioner’s experience [6].

In recent years, various machine learning algorithms, a subset of artificial intelligence and a data analysis technique that develops algorithms to predict outcomes by “learning” from data, have been investigated for early detection of sepsis-3 and outperformed than conventional or classic statistic methods, which could automatically analyze complex data and produce significant results. Following is four notable examples of such algorithms. Buchman et al. [29] concluded that machine learning-based CDS tools can accurately predict the onset of sepsis in an ICU patient 4–12 h prior to clinical recognition. Seymour et al. [30] performed different machine learning methods and suggested 4 clinical phenotypes may help in understanding the heterogeneity of treatment effects for patients with sepsis. Kashyap et al. [31] used JMP statistical software to conduct a supervised machine learning for identification of sepsis and septic shock and found it’s a reliable and efficient alternative to manual chart review. Winslow et al. [32] applied machine learning to features calculated from patient with sepsis to estimate whether or not a patient enters this pre-shock state. However, all those articles mentioned above haven’t verified the superiority of machine learning models or done relevant further analysis or offered interpretation compared to other types of prediction model. More importantly, the primary outcomes of these studies are the emergence of detection of sepsis rather than poor clinical outcomes (i.e. mortality) of sepsis. XGBoost, a decision-tree-based algorithm, has been found to be the best algorithm for machine learning and prediction competition hosted by Kaggle.com [10, 33]. Due to its best precision value and performance, XGBoost-based algorithm machine learning is increasingly emphasized as a competitive alternative to regression analysis and used in predicting clinical adverse outcomes.

In terms of the prognosis of sepsis, an artificial intelligence algorithm based on XGBoost has been published by Yuan et al. [9] in 2020. Nevertheless, both of our articles about XGboost models have its own merits. Firstly, there are several limits in Yuan’s study mentioned by himself. For instance, the features selected were according to clinical experience but not algorithm; the representativeness of features may not clear in sepsis and some important dynamic features were not included; left or right censoring may be resulted from incomplete recording of electronic medical records (EMR) when patients transfer or discharge; besides, there were no validations for the XGboost model and no traditional regression analysis was used as a control. Secondly, there are some superiorities in our model compared to Yuan’s machine learning: the features selected were according to backward stepwise analysis which increased representativeness and accuracy; some important features are not missing such as lactate, AG, etc.; data was from MIMIC-III which is an updated database and provides detailed information; classic logistic regression analysis with AUCs and DCAs were used to contrast with XGboost except for traditional scoring system; crucially, nomogram and CIC were plotted to evaluate the clinical usefulness and applicability net benefits of the model. Thirdly, of course, some common limitations exist in both of our articles: measurement bias within calculation is possible due to the method is based on experts’ opinion; sepsis could happen at any time during ICU admission (even possibly hours before labelled), although with the help of algorithm, it’s still difficult for intensivists to integrate the data of point-of-care vital signs, latest lab reports and etc. all the time and to determine the patient condition with sepsis or not according to any database.

An interesting finding in our study is that the features included in the XGBoost-based model and logistic regression model showed consistent, which indicated the excellent performances of XGBoost model were significant, although the two models may fit and perform differently in different datasets. However, these recognitions of the features and sepsis-induced mortality cannot be entirely explained. Hence, further studies and efforts are needed to investigate the mechanisms underlying the role of these variables included in patients with sepsis-3. Following is a brief summary of remarkable or controversial features included in the XGBoost model. Among these features, the weight of urine output is the greatest which represents it is the most important predictor for 30-day mortality MIMIC-III patients. This result is compatible with some clinical studies. Vieira et al. [34] reported higher urine output is associated with successful enteral nutrition therapy in septic shock patients. Laranja et al. [35] concluded that septic patients with no acute kidney injury (AKI) had a more preserved urine output compared to that in all groups with AKI or AKI/chronic kidney disease (CKD). Lin et al. [36] indicated decreased urine output could be manifested as a compensatory mechanism to maintain intravascular volume, and also imply intrinsic renal injury for patients in sepsis. Teixeira et al. [37] confirmed that the use of diuretics was inversely associated with mortality and itself may exert a protective effect. Sodium_max is an interesting feature in our XGBoost-based model. Hypernatremia can be an independent predictor of poor outcome in septic patients in the ICU, which is similar to some views [38]. However, another study [39] showed the risk of death increased by 71.6% when serum sodium was < 129 mmol/L for patients with sepsis. Lactate and AG are typical metabolic indicators. Patients with a normal lactate level alone should not be excluded life-threatening sepsis, and with high AG levels regardless of lactate levels, have high rates of mortality and should also be considered for early, aggressive therapy [40]. However, Liu and Velissaris et al. [41, 42] clearly pointed out that plasma lactate were associated with poor outcomes in patients with sepsis and predicted mortality. INR is another crucial predictive factor in the machine learning model. Several studies [43, 44] found septic patients with elevated INR and platelet count appeared to have a greater risk of death compared with those without coagulopathy. There is no doubt that age and metastatic cancer as basic demographic information could be included in the model which plays unfavorable effects for the mortality. Whereas, survival in critically ill cancer patients with sepsis improved significantly over time but reasons or mechanisms for this condition haven't been identified [45]. In consideration of the source of infection, we found blood infection ranks the highest (38.49%), followed by MRSA screen (35.49%) and urine (17.36%), which indicates that we can perform empirical antibiotics treatment, but de-escalation or determination of whether or not to stop antibiotics or successful implementation of antimicrobial stewardship may help to improve a patient's clinical prognosis while preventing adverse outcomes [46].

The strength of this study was mainly that it was the first time to predict the 30 day mortality of MIMIC-III patients with sepsis-3 using the XGBoost model, and compared to traditional regression analysis and clinical scoring system, and meanwhile verified by nomogram and CIC. We must acknowledge some other limitations of our study: firstly, because the data come from only one database and the majority of patients were white, potential bias may occur; secondly, further exploration for the database was not performed, which may lead to the abandonment of some key variables; thirdly, the proposed model was not designed to be validated by developing set from the database or our clinical data. Even so, we believe that the proposed model may contribute to further our understanding of the prognosis of patients suffering from sepsis in ICU.

## Conclusions

In conclusion, this study shows that the machine learning based on XGboost algorithm does outperform conventional logistic regressions and scoring system. This XGboost model may prove clinically useful and assist clinicians in tailoring precise management and therapy for the patients with sepsis-3 which is essential for maximizing the patient’s chance of survival.

## Supplementary information

**Additional file 1.** Extracted raw data from the MIMIC-III.

## Data Availability

The datasets used and/or analyzed during the current study are available from the corresponding author on reasonable request.

## Abbreviations

ICU: Intensive care unit
Ang-2: Angiopoietin-2
PCT: Procalcitonin
APHACHE-II: Acute physiology and chronic health evaluation-II
SAPS-II: Simplified acute physiology score-II
XGBoost: EXtreme Gradient Boosting
KDD: Knowledge Discovery and Data Mining
MIMIC-III: Medical Information Mart for Intensive Care III
BIDMC: Beth Israel Deaconess Medical Center
MIT: Massachusetts Institute of Technology
ICD-9: International Classification of Diseases and Ninth Revision
NIH: National Institutes of Health
SQL: Structure query language
BMI: Body mass index
HR: Heart rate
SBP, sysbp: Systolic blood pressure
DBP, diasbp: Diastolic blood pressure
MAP: Mean arterial pressure
TEMP: Temperature
RR, Resprate: Respiratory rate
SpO2: Oxyhemoglobin saturation
ABG: Arterial blood gas
Meanbp: Mean blood pressure
Tempc: Temperature
Bun: Blood urea nitrogen
Wbc: White blood cell
INR: International normalized ratio
Vent: Ventilation
Max: Maximum
Min: Minimum
AIC: Akaike information criterion
AUCs: Area under curves
ROC: The receiver operating characteristic curves
OR: Odds ratio
CI: Confidence interval
DCA: Decision curve analysis
CIC: Clinical impact curve
WHO: World Health Organization
SOFA: The sequential organ failure assessment
qSOFA: Quick SOFA
VTIBW: The tidal volume indexed on ideal body weight
MED: Medical-general service for internal medicine
CMED: Cardiac medical-for non-surgical cardiac related admissions
EMR: Electronic medical records
AKI: Acute kidney injury
CKD: Chronic kidney disease
